# Physico-chemical analysis of pyrolyzed bio-oil from *swietenia macrophylla* (mahogany) wood

**DOI:** 10.1016/j.heliyon.2019.e01790

**Published:** 2019-06-07

**Authors:** J.L. Chukwuneke, M.C. Ewulonu, I.C. Chukwujike, P.C. Okolie

**Affiliations:** a Mechanical Engineering, Nnamdi Azikiwe University, Awka, Nigeria; b Polymer & Textile Engineering, Nnamdi Azikiwe University, Awka, Nigeria

**Keywords:** Biomass, Bio-oil, Characterization, Pyrolysis, Renewable energy, *Swietenia macrophylla*

## Abstract

The *Swietenia macrophylla* wood used for the study was sun dried for about 48 h, pulverized using a hammer mill and then sieved to a particle size of about 425μm using Wiley milling machine. The prepared materials were pyrolyzed in a fixed-bed pyrolysis reactor in the temperature range from 425 to 500 °C. The product yields were collected at an interval of 25 °C. The maximum yield of bio-oil was recorded as 69.5wt.% at the pyrolysis temperature of 450 °C. The physicochemical properties and compositions of the feed materials and produced bio-oil were measured in order to quantify their potential for bio-energy use and industrial applications. The properties specifically measured include density, moisture content, ash content, pH, refractive index, cetane index, elemental composition, viscosity, and heating values. The ultimate analysis of the product showed that the contents of carbon, oxygen, hydrogen, nitrogen, and sulfur were 50.2%, 42.6%, 6.6%, <0.4% and <0.06% respectively. The viscosity, density, pH, moisture content, API gravity, ash content, HHV and LHV of bio-oil produced were found to be 4.6 mm^2^/s, 0.951 g/ml, 5.64, 21.4wt.%, 19.29, 0.15wt%, 29.52 MJ/kg and 28.08 MJ/kg respectively. These values were found to be in the ranges of values reported in the literature for bio-oils produced from biomass. The produced bio-oil had the much needed organic compounds typical of other woody biomass employed in commercial bio-oil manufacture. These compounds were classified into several groups; organic acids, ketones, phenols, alcohols, and aldehydes. The main components identified in the bio-oil are the aromatic and aliphatic compounds as well as carboxyl groups. The GCMS analysis of the product indicated the presence of 24 compounds which are useful as industrial chemicals and flammable gases: they include alkanes, alkenes, phenols, hydrogen, and levoglucosan. This study on bio-oil has demonstrated that mahogany wood is a useful biomass for the much sort potential fossil fuel substitute and finds vast application in the biofuel industry.

## 1 Introduction

Energy is a prime mover in the development of the world economy. Fossil fuels are major players in the world's total energy needs. Energy demand is growing everyday with the swift outgrowth of the economy and the population (Raquel et al., 2018; Sharma and Sheth, 2015). The negative impact caused by the burning and use of fossil fuels for different purposes has created an environmental danger owing to the emission of greenhouse gases (Hasan et al., 2011; Mohamed, 2018), climate change, reduction in fossil fuel reserves (Sonil et al., 2014), acid rain and global warming. The result is an increased quest for renewable resources worldwide (Goyal et al., 2008; Sareekam et al., 2016). The exploit of biomass as a basis of fostering energy abundance and increased renewable options has attracted a lot of interest (Zhao and Li, 2016; Chin et al., 2015). In view of current energy scenarios, biomass presents potential as eco-friendly alternative source of renewable energy which is accessible through diverse biological, physical and thermal processes (Hossain et al., 2014; Swenson, 2016).

Biomass (organic material) can be acquired from forests, agricultural wastes, household wastes, plantations and industries. Hydrocarbon compounds contained in biomass could yield heat energy producing fuels (Fardhyanti and Damayanti, 2017; Montevecchi, 2016). Biomass holds promise as a good feedstock for generating liquid fuels in the imminent future (Demiral and Ayan, 2011; Ilknur et al., 2012; Donate, 2014; Joardder et al., 2015). Biomass particles can be converted into solid char, gases, and condensable pyrolytic vapour as advanced merit fuels (Witchakorn, 2015; Rahmat et al., 2015). However, at atmospheric pressure, pyrolysis can directly generate liquid fuels in the absence of oxygen in a reactor at temperature ranges of 400–600 °C.

Pyrolysis is the most commonly used method of thermo-chemical conversion for the transformation of biomass residues into bio-oil and bio-char (Josilaine et al., 2013; Xueyong et al., 2017; Oyebanji and Ololade, 2017). Fast pyrolysis is the process of heating an organic material (e.g. wood) at high temperature in the range of 400–600 °C and short reaction time, in the absence of an oxidizing agent (Mohammad et al., 2014; Puttiphon and Sukritthira, 2014; Mazlan et al., 2015a, b; Narayan et al., 2017; Meheretu et al., 2019). Fast pyrolysis requires small particle size due to fast heating which informs the nature of bio-oil produced (Mazlan et al., 2014; Hyeon et al., 2010; Wasakorn et al, 2017). It could be deduced that pyrolyzing waste materials has a substantial economic value and could foster a cleaner environment (Nhlanhla and Edison, 2014; Kanyaphorn and Tanongkiat, 2015). The pyrolysis bio-oil produced using renewable lignocellulosic biomass in fast pyrolysis can be seen as the most economical liquid fuel generated from organic materials today (Liu et al., 2014; Kiky et al., 2015; Xueyong et al., 2017).

Bio-oil produced by pyrolysis is a dark-brown organic liquid with a burly bitter smell (Faisal et al., 2011; Wenfei et al, 2018) and it typically possesses lower hydrogen and carbon contents and high oxygen content when compared with fossil fuels (Josilaine et al., 2013; Stefanidis et al., 2013; Joardder et al., 2015). It is thermodynamically an unbalanced liquid but wealthy in functional groups (Josilaine et al., 2013). Bio-oil, when subjected to combustion tests, has high burning effectiveness which makes it a viable fuel (Josilaine et al., 2013). Different biomass stock would give varying yields and characteristics of pyrolyzed oil.

For the purpose of this study, fast pyrolysis process was employed to extract bio-oil from mahogany (*Swietenia macrophylla)* wood wastes which are among the most widely available biomass in the world (Maria et al., 2016; Bridgwater, 2018; Rahmat et al., 2015; Ogunsannwo et al., 2014). The pyrolysis was done at various yield temperatures followed by subsequent analysis of the products.

## 2 Materials and methods

### 2.1 Biomass sample preparation

The Mahogany (*Swietenia macrophylla)* wood waste sample used was sourced from a local market mill in Enugu, Nigeria. The sample was sun dried for about 48 h, pulverized using a hammer mill and then sieved to a particle size less than 1mm passing mesh 40 (425μm) using Wiley milling machine.

### 2.2 Pyrolysis process

**Fig. 1:**
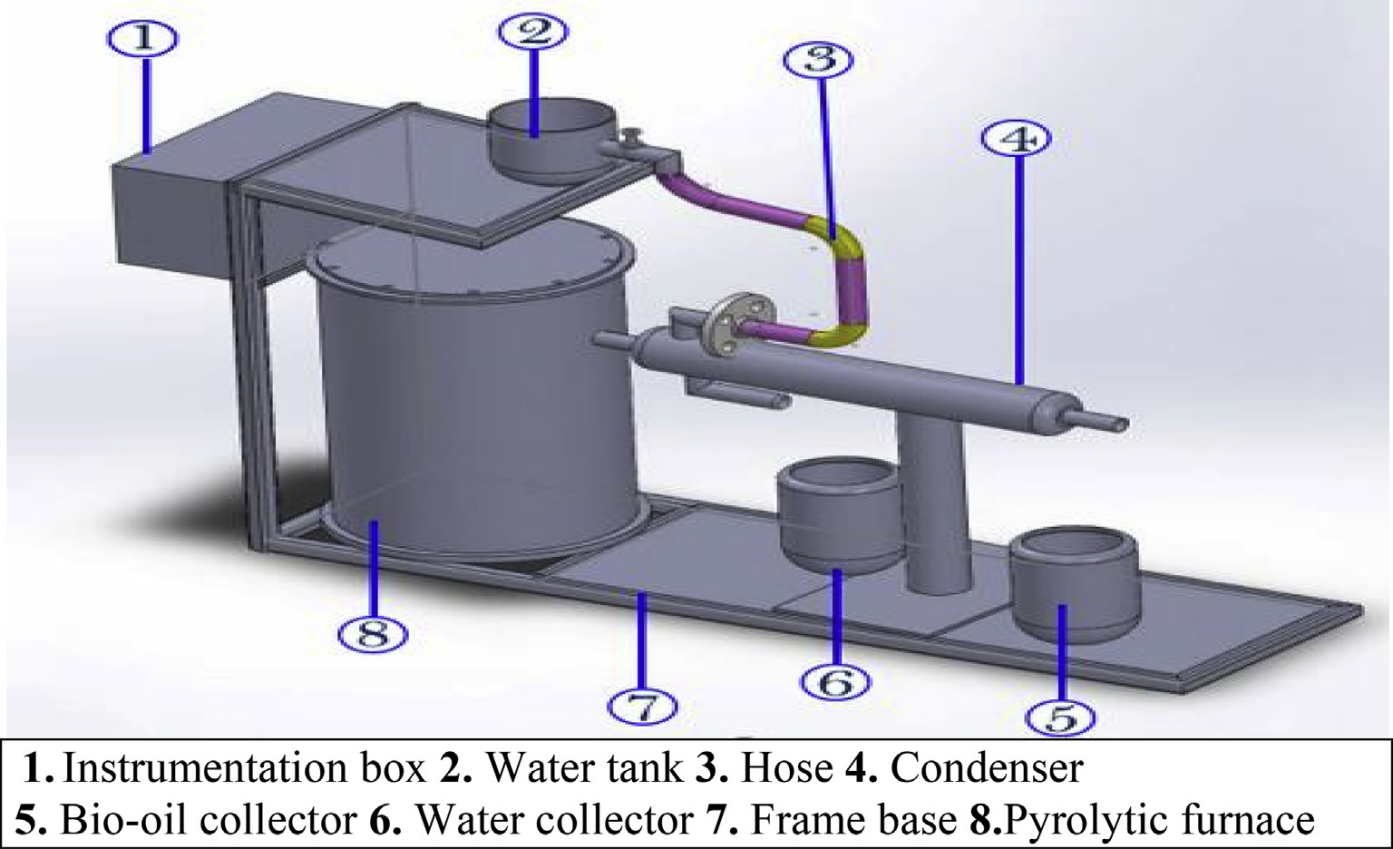
Schematic diagram of reactor unit.

Bio-oil was produced from mahogany waste wood biomass feedstock using a fixed bed reactor pyrolysis system (at the Mechanical Engineering Laboratory, Nnamdi Azikiwe University, Awka, Nigeria) with a capacity of 1 kg/h, at the atmospheric pressure, see Fig. 1. Pyrolysis process procedures used are as described by Suyati et al. (2015) and Chukwuneke et al, (2016). The pulverized mahogany wood waste sample (prepared as described above) was weighed. 1 kg/h of the sample was heated in a pyrolysis reactor by an electric heater at a heating rate of 500 ^°^C/s with a pyrolysis product residence time of <1.5s. The temperature was controlled by an external PID controller and measured by a Ni–Cr–Ni thermocouple fixed inside the bed. The reaction was executed at temperatures ranging from 425 to500 °C at an interval of 25 °C. The ensuing gas was passed through a condenser to obtain an oil-water mixture which was automatically separated into bio-oil and water. The bio-oil was there after stored at 4 °C in a refrigerator (Guizani et al., 2017).

### 2.3 Characterization of bio-oil

The pale brown bio-oil obtained from biomass by pyrolysis reaction which was stored in a refrigerator was thereafter characterized. The density, moisture content, ash content, pH, refractive index, cetane index, elemental composition, viscosity, and heating values as suggested by Hasan et al. (2011) were measured. The chemical constituents of the bio-oils were determined by GC/MS analysis while the organic functional groups in the oils were characterized via FTIR techniques.

A density measurement bottle (pycnometer) was used to obtain the density of bio-oil. Viscosity measurement of the bio-oil was performed using a U-tube viscosity (PSL ASTM-IP 350) from which the kinematic viscosity at 40 °C was determined. The pH measurement was carried out with a digital pH meter (Hanna model-HI 8424). Moisture content in the oil was measured by Karl-Fischer titration method in accordance with ASTM E203 standards at PRODA laboratory, Enugu, Nigeria. The solid content in the pyrolyzed bio-oil was measured as ethanol insoluble portion (Suchithra et al., 2014; Dufourny et al., 2019). The acid value of the oil was determined by direct titration with standard potassium hydroxide solution according to Anyanwu and Adefila, 2014. The ash content was determined according to ASTM D3174-07. Refractive index was measured using a digital tabletop refractometer (Hanna model-HI 96800).

The elemental analysis is necessary to ascertain the Carbon, Hydrogen, Oxygen, Nitrogen and Sulfur contents of the oil sample. Carbon, Hydrogen and Nitrogen contents of the feedstock were determined using LECO CHN 2000 Elemental Analyzer according to the test methods of ASTM D5291-02 while the Oxygen content was determined by difference. Sulfur content was determined using LECO SC-432DR (trace EI 6-2A) according to the standard procedure of ASTM D4239-83. The heating value of the oil was obtained according to ASTM D5865 standard test method for Gross calorific value of Coal and Coke on a dry basis for sun-dried samples. The PAAR 1341 oxygen bomb calorimeter with benzoic acid pellets was used to determine the Gross calorific value (HHV) while the net calorific value (LHV) was calculated using wt.% of Hydrogen resulting from elemental analysis of the sample.

The GC-MS of the oil obtained from the feedstock was analyzed with an Agilent 7890 GC/5975MS using a DB-1701 column for identification and quantification of their chemical compositions while the FTIR spectra of the bio-oil and their aromatic, aliphatic and polar sub-fractions were obtained with Perkin-Elmer 100 model FTIR system spectrum GX. The bio-oil obtained at the maximum oil yield condition was tested for its fuel properties using the ASTM standard method for petroleum products (Ilknur and Emine, 2011). These include flash point, fire point, pour point, cloud point, cetane index etc.

## 3 Results and discussion

### 3.1 Physical and chemical properties

The colour of the pyrolyzed wood bio-oil from *Swietenia Macrophylla* (Mahogany) was pale brown. The elemental composition, moisture content, density, viscosity, ash content, pH, HHV and LHV of the feed materials and bio-oil are given in Tables 1 and 2.

**Table 1:** Properties of mahogany wood Waste Sample.

Ultimate Analysis		Proximate Analysis	
Component	Content (wt.%)	Component	Content (wt.%)
C	55.30	Moisture	5.80
H	4.56	Volatile Matter	79.11
N	<0.34	Fixed Carbon	13.85
O	39.26	Ash	1.24
S	<0.60		
HHV (MJ/kg)	21.26	LHV (MJ/kg)	20.27

**Table 2:** Properties of pyrolysis bio-oil.

Physical Properties	Typical Value
Moisture Content (wt.%)	21.4
Kinematic viscosity @ 40 °C (mm^2^/s)	4.6
pH	5.64
API gravity	19.29
Elemental Analysis (wt.%) C H O N S Ash	50.2 6.6 42.6 <0.4 <0.06 0.15
HHV (MJ/kg) LHV (MJ/kg)	29.52 28.08
Density (g/ml)	0.951

The results of Table 1 show that mahogany wood waste sample has high energy content and therefore is suitable for the production of bio-oil. From Table 2, the results show that the bio-oil produced in this study contains 50.2% carbon and 42.6% oxygen. The low sulfur (<0.06%) and Nitrogen (0.4%) contents are indicative of the fact that the bio-oil has a low pollutant effect which implies that it is eco-friendly. The values of HHV and LHV are 29.52 mJ/kg and 28.08 mJ/kg respectively which are lower compared with other viable fossil fuels probably due to the high moisture content of the bio-oil. The above heating values however, are greater than the values obtained for other bio-oils by Okoroigwe et al. (2015) and Hossain et al. (2014) but of the same range of values as reported by Oyebanji et al. (2017). The moisture content and viscosity of the oil determined from this work do not differ significantly from those reported by Suchithra et al. (2010) and Okoroigwe et al. (2012). The pH and ash content are close to the range for bio-oils from biomass (Okoroigwe et al., 2012; Kato et al., 2016; Oyebanji et al., 2017) and in the same range as those reported by Suchithra et al. (2010). The pH level of this bio-oil is nearly neutral but could be neutralized in other to prevent corrosion and other reactions of the bio-oil when used in engines and boilers (Onal et al., 2017; Okoroigwe et al., 2012). The moisture contentin the wood is reported to be high and may cause saponification and hydrolysis in the bio-oil production (Joseph et al., 2014). Saponification value of 235.07mgKOH/g was recorded from the oil. The density of the oil from *Swietenia Macrophylla* wood waste is 0.951 g/cm^3^ which meets the density specifications for engine use (Renato et al., 2016; Valente et al., 2011). The relative density was established to be within the range of values reported for biodiesel fuels (Joseph et al., 2014). The lower the density, the heavier the fuel and the harder it is to burn.

### 3.2 Product yield

**Fig. 2:**
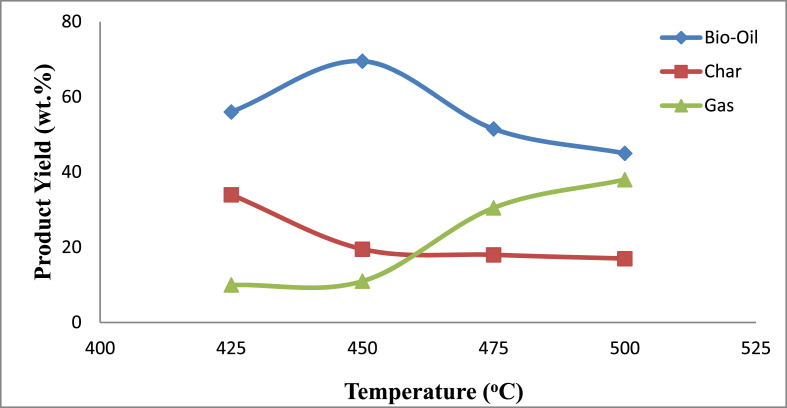
Variation of bio-oil yield with pyrolysis temperature.

The effect of temperature on the pyrolysis products yields (bio-oil, char and gas) of mahogany chip wood was determined and plotted in Fig. 2. Pyrolyzing mahogany wood chips at 425 °C gave 56wt.% bio-oil. Increase in the pyrolysis temperature to 450 °C had the bio-oil yield increase to a maximum yield of 69.5wt.% but further increase in temperature showed a net decrease of oil yield. As the pyrolysis progressed, the char yield decreased from about 34wt.% to 17wt.% with increase in pyrolysis temperature. It was observed that the gas yield increased from about 10wt.% at 425 °C to 38wt.% at 500 °C as pyrolysis temperature increased.

These results are comparable with those reported in the literature (Oyebanji and Ololade, 2017; Okoroigwe et al., 2012) as shown in Table 3. The maximum yield of 69.5wt.% for bio-oil obtained in this study is lower than that reported by Okoroigwe et al. (2012) by about 2% but higher than others listed on Table 3. The large value of the maximum bio-oil yield may be attributed to the secondary reactions of the compounds (heavy molecular weight) in the pyrolysis vapours, which are activated at temperatures over 500 °C (Hyeon et al., 2010; Giuffre et al., 2017; Chuxia et al., 2019). The peak temperature of 450 °C for oil yield is marginally lower than the usual range of 500–520 °C for wood biomass as reported by Guedes et al. (2018) and Hyeon et al. (2010).

**Table 3:** Comparison of woody biomass materials.

SN	Materials	Bio-oil Yield wt.%	Processing Conditions	Temperature ^o^C	References
1	Mahagony (*swieteniamacrophylla*) wood waste	69.5	Fixed bed-fast pyrolysis	450	This study
2	TectonaGrandis wood	61	Fixed bed-fast pyrolysis	450	Oyebanji and Ololade (2017)
3	Waste furniture sawdust	58.1	Fluidized bed-fast pyrolysis	450	Hyeon et al. (2010)
4	Pine wood	50	Auger reactor fast pyrolysis	450	Suchithra et al. (2010)
5	Meranti wood sawdust	30	Fixed bed-fast pyrolysis	550	Mazlan et al. (2015)
6	Ogbono wood	60	Fixed bed-fast pyrolysis	-	Okoroigwe et al. (2015)
7	Mango wood	61	Fixed bed-fast pyrolysis	-	Okoroigwe et al. (2015)
8	Neem wood	66	Fixed bed-fast pyrolysis	-	Okoroigwe et al. (2015)
9	Ogirisi wood	57	Fixed bed-fast pyrolysis	-	Okoroigwe et al. (2015)
10	Tropical almond wood	53	Fixed bed-fast pyrolysis	-	Okoroigwe et al. (2015)
11	GmelinaArborea wood	71	Fixed bed-fast pyrolysis	454	Okoroigwe et al. (2012)

The ASTM standard method for petroleum products was employed in testing the fuel properties of the pyrolysis oil derived at the maximum oil yield conditions. The measured parameters include; fire point, flash point, cloud point, pour point, cetane index, aniline point, etc. as shown in Table 4.

**Table 4:** Fuel properties of bio-oil.

SN	Properties	Values
1	Flash Point (^°^C)	68.0
2	Fire Point (^°^C)	72.0
3	Pour Point (^°^C)	13.5
4	Cloud Point (^°^C)	19.5
5	Aniline Point (^°^C)	22.5
6	Cetane Index	38.9

### 3.3 Chemical analysis of the bio-oil

#### 3.3.1 GC-MS spectrometry analysis

**Fig. 3:**
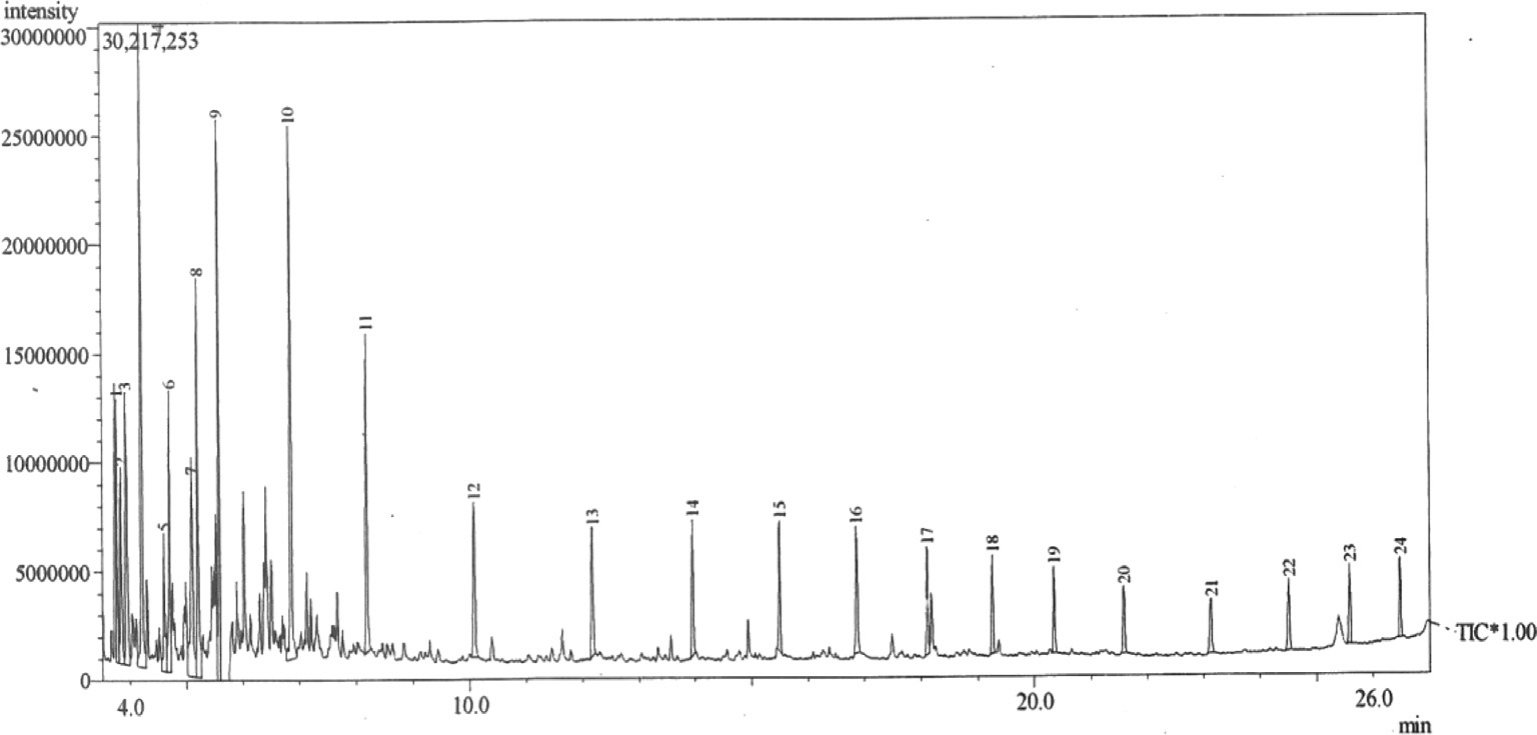
GC-MS of bio-oil components produced from pyrolysis of *S. macrophylla* wood.

The chromatograms of the GC-MS analysis of *Swietenia Macrophylla* wood are presented in Fig. 3 while the twenty-four (24) chemical compounds obtained from the GC-MS analysis of the bio-oil are presented on Table 5.

**Table 5:** Chemical composition of bio-oil from *S. Macrophylla* wood on chromatograms.

Compounds	R_T_	R_index_	Area (%)	Height (%)
Methylbenzene (Toluene)	3.768	794	4.40	5.11
2,4-methylhexane	3.842	752	3.48	3.81
3-methylheptane	3.842	752	1.52	2.05
1,4-dimethylcyclohexane	3.933	842	5.99	5.30
2-methyldecane	4.208	1051	11.58	12.51
2,6-dimethylheptane	4.600	788	2.79	2.67
Trans-1-ethyl-3-methylcyclopentane	4.700	821	5.02	5.47
1,1,2,2-tetramethylcyclopropane	4.700	629	2.55	2.47
Dicyclohexyl ester	4.700	880	1.55	1.38
Ethylcyclohexane	4.700	850	1.53	1.06
2,3,4-trimethylhexane	5.092	724	6.27	4.24
5-(1-methylethylidene)	5.200	824	8.05	7.74
2-methylnonane	5.575	916	8.23	10.91
2,7-dimethyloctane	6.850	887	10.55	10.36
5-methyldecane	8.183	1051	5.90	6.20
2-methyltridecane	10.075	1150	3.67	3.00
4,8-dimethyltridecane	12.175	1313	2.83	2.51
4,8-dimethyltridecane	13.950	1384	2.75	2.65
2,6,11-triethyldodecane	18.108	1320	2.13	2.09
2-methylnonadecane	20.350	1945	1.75	1.68
2-butyl-1-octanol	21.583	1393	1.58	1.31
Ether, 2-ethylhexylvinyl	21.583	1017	2.53	2.52
Tridecanol, 2-ethyl-2-methyl-	25.583	1770	1.52	2.02
2,6,10,15-tetramethyl-heptadecane	25.583	1852	1.83	1.91

From Table 5 and Fig. 3, the most abundant components are, 2-methyldecane (11.58%) and 2,7-dimethyloctane (10.55%). The other major compounds present in the bio-oil are 5-(1-methylethylidene) (8.05%), 2-methylnonane (8.23%), 1,4-dimethylcyclohexane (5.99%), 5-methyldecane (5.90%), 2,3,4-trimethylhexane (6.27%), trans-1-ethyl-3-methlcyclopentane (5.02%), methylbenzene (4.40%), 2-methyltridecane (3.67%), 2,4-dimethylhexane (3.48%), 4,8-dimethyltridecane (5.58%), 2,6-dimethlheptane (2.79%). The GC-MS results indicate the presence of hydrocarbons, fatty acids, alcohols, esters, ethers, phenolic and ketone compounds confirming that it can be effectively used as biodiesel (ester and Alkanes) and in Pharmaceutical and dyes industries (fatty acids: linoleic acid and tetradecanoic acid). The chemical compounds are comparable to those identified by some researchers working with other woody biomass class (Kim et al, 2017; Okoroigwe et al., 2012; Oyebanji and Ololade, 2017; Oyebanji et al., 2017).

#### 3.3.2 FT-IR spectrum analysis

**Fig. 4:**
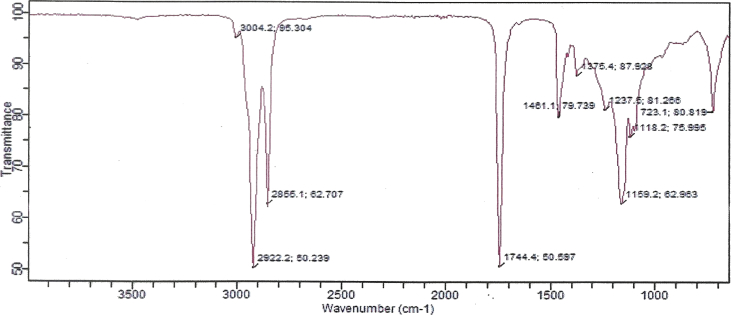
Ftir spectrum of bio-oil from pyrolysis of *Swietenia Macrophylla* wood.

The functional group compositions present in the bio-oil from *Swietenia Macrophylla* wood chips were identified at the wavelength range between 4000 – 650 cm^−1^in the FT-IR Spectrum analysis as shown in Fig. 4. The possible functional group compositions and possible compounds are tabulated in Table 6.

**Table 6:** Functional group compositions of bio-oil from *Swietenia Macrophylla* wood.

Functional Group	Wavelength (cm-1)		Molecular Motion
	Range	Actual	
Aromatics Carboxylic Acids	3020–3000 3400–2400	3004.2	C–H stretch O–H stretch
Alkanes	2950–2800	2922.2 2855.1	C–H stretch
Esters Aldehydes	1750–1735 1775–1740	1744.4	C=O stretch
Nitro groups	1550–1490 1390–1300	1461.1 1375.4	–NO_2_ (aromatic) –NO_2_ (aliphatic)
Alcohols Esters	1260–1000 1260–1230	1237.5	C–O stretch C–C(O)–C stretch (acetates)
Amines Ketones	1200–1025 1300–1100	1159.2 1118.2	C–N stretch (alkyl) C–C stretch
Alkyl halides	785–540	723.1	C–Cl stretch

The presence of a broadband corresponding to 3004.2cm^−1^was assigned to = C–Hstretching of the C–C double bonds. A broad absorption band observed between 3400 – 3000cm^−1^ is credited to the O–H stretching vibrations of hydroxyl groups from phenols, alcohols, and carboxylic groups bond to aromatic rings. The peak value between 2950 and the 2800cm^−1^ region is assigned as stretching of C–H saturated bonds suggesting the presence of alkanes. The band absorbance at 1744.4cm^−1^ could be caused by C=O stretching groups probably from aldehydes and esters. The observed peak at 1461.1cm^−1^ shows the presence of C=C stretching vibration form of aromatic compounds, while the deformation vibration at 1375.4cm^−1^ indicates the presence of aliphatic CH_3_. The band in the region from 1260 -1230cm^−1^ shows the possible presence of esters in the C–O stretching group. The absorbance peak at 1237.5cm^−1^ could be assigned to the aromatic CO– and phenolic–OH stretching, while the peak at 1159.2cm^−1^ corresponds to the stretching of aliphatic ether C–O or alcohol C–O, and the peak at 1118.2cm^−1^ indicates the presence of C–N stretch (alkyl) containing compounds as amines. The 723.1cm^−1^ as C–H bonds indicates the presence of another group of aromatic compounds considering the band between 785–540cm^−1^ of the C–H stretch group. Majority of the functional groups have been identified by researchers working with other woody biomass classes (Kumar et al., 2010; Pinto et al., 2018; Ogunsanwo et al, 2014) and their classifications are similar to those reported in this study.

## 4 Conclusion

In this paper, pyrolysis of *swietenia macrophylla* wood chips was conducted at selected temperatures ranging from 425 °C to 500 °C and particle size of less than 425μm in bed reactor and the oil produced was characterized. The maximum oil yield of 69.5wt.% was achieved at a temperature of 450 °C which is reasonably high when compared with the yield of bio-oil from other energy classified woody biomass materials. The measured physio-chemical properties of bio-oil produced from *swietenia macrophylla* wood are comparable with those obtained from liquid products from other woody biomass materials. Furthermore, the oil produced from *swietenia macrophylla* wood chips gave high carbon, oxygen and hydrogen contents. The HHV of 29.52 mJ/kg is close to the commercial biodiesel products requirement. The bio-oil produced contains a vast range of functional groups of alkanes, aliphatic, aromatic, aldehydes, ketones, alcohols, carboxylic acids, phenols and the majority of the functional groups indicate the presence of oxygen. The GCMS analysis of the bio-oil shows that the woody biomass may possibly be a source of useful energy (ester and Alkanes) and industrial chemicals (linoleic acid and tetradecanoic acid). It is possible that improved fuel quality can be acquired from the bio-oil product if upgraded for future renewable energy demands.

## Declarations

### Author contribution statement

Jeremiah Chukwuneke: Conceived and designed the experiments; Performed the experiments; Wrote the paper.

Chinomso Ewulonu, Iheoma Chukwujike & Paul Okolie: Analyzed and interpreted the data; Contributed reagents, materials, analysis tools or data.

### Funding statement

This work was supported by Nnamdi Azikiwe University, Awka, Nigeria through the 2012–2014 Merged TETFUND Research Project Intervention Funds 8th Batch.

### Competing interest statement

The authors declare no conflict of interest.

### Additional information

No additional information is available for this paper.
